# HBV-Specific TCR–T Cell Therapy Combining mRNA Electroporation and Lentiviral Transduction: Treatment Regimen for Recurrent HBV-Related HCC after Liver Transplantation

**DOI:** 10.1158/1078-0432.CCR-25-1245

**Published:** 2025-07-24

**Authors:** Qiang Zhao, Jinbo Huang, Weixin Luo, Haidong Tan, Regina Wan Ju Wong, Zhiying Liu, Meiting Qin, Jiahao Li, Sarene Koh, Lu-En Wai, Tingting Wang, Jia Dan, Zhiyong Guo, Xiaoshun He

**Affiliations:** 1 Organ Transplant Center, The First Affiliated Hospital, Sun Yat-sen University, Guangzhou, China.; 2 Guangdong Provincial Key Laboratory of Organ Donation and Transplant Immunology, Guangzhou, China.; 3 Guangdong Provincial International Cooperation Base of Science and Technology, Guangzhou, China.; 4 Guangzhou Red Cross Hospital, Guangzhou, China.; 5 Second Department of Hepatopancreatobiliary Surgery, China-Japan Friendship Hospital, Beijing, China.; 6 Lion TCR Pte Ltd, Singapore, Singapore.; 7 Zhongshan School of Medicine, Sun Yat-sen University, Guangzhou, China.

## Abstract

**Purpose:**

This study aimed to preliminarily evaluate the safety, tolerability, and antitumor efficacy of hepatitis B virus (HBV)-specific T-cell receptor (TCR)–T cell therapy combining mRNA electroporation and lentiviral transduction in patients with recurrent HBV–hepatocellular carcinoma after liver transplantation.

**Patients and Methods:**

In this pilot study (NCT04677088), two types of autologous HBV-specific TCR-redirected T cells were assessed without prior lymphodepletion: (i) multiple infusions of mRNA-electroporated HBV–TCR–T cells (mRNA–HBV–TCR–T cells) and (ii) one to three infusions of lentiviral-transduced HBV–TCR–T cells (lenti-HBV–TCR–T cells). Treatment-related adverse events were assessed using the Common Terminology Criteria for Adverse Events, and antitumor efficacy was evaluated using CT imaging according to RECIST v1.1 criteria. Progression-free survival (PFS) was defined as the time from the start of study treatment until objective tumor progression or death.

**Results:**

Both mRNA-electroporated and lentiviral-transduced HBV-specific TCR–T cells demonstrated a favorable safety profile, with only grade 1 to 2 treatment-related adverse events observed. In the mRNA–HBV–TCR–T cells cohort, the median PFS was 2.32 months (range, 1.87–2.77 months). The combination therapy cohort (mRNA–HBV–TCR–T cells + lenti-HBV–TCR–T cells) showed a median PFS of 7.34 months (range, 4.47–7.60 months). CT imaging indicated effective tumor control in the combination therapy group.

**Conclusions:**

This study preliminarily suggests that the combination of mRNA–HBV–TCR–T cells and lenti-HBV–TCR–T cells could be a safe and potentially effective approach for treating patients following liver transplantation in the context of lifelong immunosuppression drug administration. Further studies are needed to refine treatment strategies and assess long-term safety and efficacy in this special patient population.

**Translational Relevance**

In this pilot study (NCT04677088), the safety and efficacy of hepatitis B virus (HBV)–T-cell receptor (TCR)–T cell therapy were evaluated in six patients with recurrent HBV–hepatocellular carcinoma after liver transplantation. Two types of autologous HBV-specific TCR-redirected T cells were assessed without prior lymphodepletion: (i) multiple infusions of mRNA–HBV–TCR–T cells and (ii) one to three infusions of lentiviral-transduced HBV–TCR–T cells (lenti-HBV–TCR–T cells). Both cell types demonstrated a favorable safety profile, with no treatment-related serious adverse events observed. HBV-specific TCR–T cell therapy, utilizing both mRNA electroporation and lentiviral transduction, may offer a potentially effective treatment strategy for patients with recurrent HBV-related hepatocellular carcinoma after liver transplantation.

## Introduction

Hepatocellular carcinoma (HCC) is the most prevalent type of primary liver cancer in adults and remains a leading cause of cancer-related mortality worldwide (1). Chronic hepatitis B virus (HBV) infection is a major etiologic factor, accounting for approximately 50% of global HCC cases (2). Following the progression from hepatitis to cirrhosis and ultimately to cancer, liver function in patients with HCC often deteriorates to an irreversible terminal stage, rendering liver transplantation (LT) the only effective curative treatment. However, postoperative tumor recurrence occurs in 40% to 60% of patients whose preoperative tumor burden exceeds the Milan criteria, leaving limited therapeutic options (3). Posttransplant recurrence (PTR) is typically characterized by widespread metastases, involving multiple organs such as the lungs, liver, bones, lymph nodes, and adrenal glands in more than 50% of cases. This condition is associated with poor outcomes, with a median survival of less than 1 year following diagnosis (4). Long-term remission of PTR is rare, and currently, there is no standard-of-care drug for recurrent liver cancer after LT, underscoring the urgent need for innovative therapeutic approaches to manage recurrent HBV–HCC in LT recipients.

Adoptive cell therapy, including chimeric antigen receptor T-cell (CAR-T) therapy and T cell receptor-engineered T-cell (TCR-T) therapy, has emerged as a promising strategy in cancer immunotherapy (5). Whereas CAR-T therapy has demonstrated significant efficacy in hematologic malignancies, its effectiveness in solid tumors has been limited (6). In contrast, TCR-T therapy has shown better outcomes in clinical studies of solid tumors due to its higher sensitivity and broader antigen recognition (7). The first TCR-T therapy for treating solid tumor was approved by the US FDA in 2024 (8). The success of TCR-T therapy depends on the appropriate selection of target antigens, which can be broadly categorized into three types. Tumor-associated antigens, commonly expressed on both tumor and normal tissues, carry a risk of off-target toxicity (9, 10). Tumor-specific neoantigens, encoded by mutated tumor cell genes, offer higher specificity but are limited in applicability because of tumor heterogeneity (11). Viral antigens, particularly in virus-associated tumors, represent an ideal target, as they provide superior immunogenicity, specificity, and universality (12). Consequently, TCR-T cells specifically targeting HBV antigens hold significant potential for the treatment of HBV–HCC (13). The introduced HBV-specific TCRs, comprising α and β chains linked by disulfide bonds, engage endogenous signaling pathways upon recognizing HBV epitopes presented by HCC cells, initiating cytotoxic proteins, cytokine production, and tumor cell killing.

The safety and potential efficacy of autologous mRNA-electroporated HBV–TCR–T cells in patients with advanced primary HBV–HCC have been demonstrated (13–17). The use of functionally transient mRNA TCR-T cells offers a safer therapeutic strategy by limiting prolonged TCR expression, thereby reducing the potential for sustained “on-target–off-tumor” liver toxicity (18). This is particularly important for patients with primary HBV–HCC, in whom the risk of targeting normal HBV-infected hepatocytes remains a safety concern. Given that liver function in these patients may already be compromised, preserving liver function and preventing further damage are also critical considerations. However, its short duration of TCR expression may necessitate repeated administrations to achieve therapeutic efficacy (19).

In contrast, in the setting of recurrent HCC after LT, the HLA mismatch between the transplanted liver and the patient’s native immune system makes long-term TCR expression unlikely to cause significant liver toxicity. This is because HBV–TCR–T cells selectively target HBV–HCC tumors in non-HBV–infected organs rather than HBV-infected liver. Moreover, preserving the functionality of TCR–T cells is critical for their clinical efficacy, particularly in the immunosuppressive environment following LT. A major challenge in implementing immunotherapy for organ-transplanted recipients is that posttransplantation immunosuppression regimens may compromise the efficacy of T-cell therapy. In the case of LT, maintenance immunosuppression regimens typically started with tacrolimus alone or in combination with mycophenolate mofetil after transplantation continue lifelong. These drugs were designed to broadly suppress the T cell–mediated immune responses and prevent graft rejection. It has been demonstrated that even short-term exposure to these immunosuppressive drugs significantly impairs the function and migration of T cells (16, 20).

Stable genetic modification approaches, such as retroviral and lentiviral transductions, have been widely applied in adoptive T-cell therapy for both hematologic malignancies and solid tumors. It has been demonstrated that in patients with recurrent HCC after LT, retroviral-transduced HBV–TCR–T cells could induce systemic IFN-γ and IL-8 release even at a low dose of 1.2 × 10^4^ cells/kg in a single administration with manageable safety (14). A key safety advantage of using lentiviral-transduced HBV–TCR–T cell therapy in patients with LT is that, due to common HLA mismatches between the transplanted liver and the recipient, HBV–TCR–T cells selectively recognize HBV antigens presented on the patient’s own HLA class I molecules. This selectivity reduces the risk of targeting hepatocytes in the transplanted liver, thereby minimizing the potential for liver injury associated with prolonged TCR expression following HBV–TCR–T cell infusion.

However, the use of viral vectors for stable genetic modification poses concerns about persistent immune activation, which could increase the risk of chronic inflammation and, potentially, organ rejection in transplant recipients. This raises the critical question of whether the long-term persistence of HBV–TCR–T cells following viral transduction presents an acceptable risk–benefit profile in this patient population. Additionally, the need for lymphodepleting chemotherapy prior to TCR–T cell infusion introduces further safety considerations such as cytopenia, increased infection risk, and delayed immune reconstitution, all of which must be closely monitored in these immunocompromised patients (21). In contrast, mRNA–HBV–TCR–T cells do not require prior lymphodepletion, thereby avoiding these side effects and offering a safer alternative. 

Despite these challenges, evaluating the feasibility of stable HBV–TCR expression remains essential, as extending patient survival is the gold standard in cancer treatment. This is particularly urgent for patients with recurrent HBV–HCC after LT, in whom treatment options remain extremely limited due to the lack of effective standard-of-care therapies approved by regulatory agencies worldwide. In this study, HLA-matched TCRs targeting HBV surface antigen (HBsAg) or core antigen (HBcAg) were selected from a library of HBV-specific TCRs restricted by different HLA class I molecules (provided by Lion TCR; ref. 22). These TCRs were introduced into autologous T cells using two distinct approaches either via mRNA electroporation to generate transiently expressing mRNA–HBV–TCR–T cells or via lentiviral transduction to produce stably modified lenti-HBV–TCR–T cells.

Patients were enrolled into three cohorts (*N* = 2 in each cohort, a total of six patients). Patients in cohort 1 received multiple infusions of mRNA-electroporated T cells (mRNA–HBV–TCR–T cells). Following the confirmation of its safety, cohort 2 patients received multiple mRNA–HBV–TCR–T cells followed by lentiviral-transduced T cells (lenti-HBV–TCR–T cells). Cohort 3 patients received a single infusion of mRNA-HBV–TCR–T cells followed by lenti–HBV–TCR–T cells. This study evaluates the safety profiles of HBV–TCR–T cell therapies in patients with recurrent HBV–HCC after LT and explores its therapeutic efficacy.

## Patients and Methods

### Study design and patients

In the current pilot study, we examined the safety and efficacy of HBV–TCR–T cell therapy using mRNA-electroporated HBV–TCR–T cells (mRNA–HBV–TCR–T) and lentiviral-transduced HBV–TCR–T cells (lenti-HBV–TCR–T) in six patients with HBV–HCC with tumor recurrence after LT at the First Affiliated Hospital of Sun Yat-sen University. The study was approved by the respective Institutional Review Board of the First Affiliated Hospital, Sun Yat-sen University, and complied with the Declaration of Helsinki. Written informed consent was obtained from all patients.

The main inclusion criteria for patients were as follows: (i) patients ages ≥18 years who had undergone orthotopic LT; (ii) patients who had undergone LT for more than 4 weeks and had fully recovered from the surgery procedure of LT; (iii) patients with histologic or cytologic diagnosis of HCC prior to transplantation; (iv) patients diagnosed with recurrence of the original tumor lesion or metastatic tumor lesion after LT; (v) patients with HBV infection history, with positive serologic HBsAg testing, or detectable HBV DNA or HBV RNA in the peripheral blood; and (vi) patients whose liver and kidney functions met the following criteria: (a) Alanine transaminase (ALT) and aspartate aminotransferase (AST) should be within 2.5 times the upper limit of reference values. If there was abnormal liver function due to tumor metastasis, ALT and AST should be within five times the upper limit of reference values; (b) total bilirubin (TBIL) ≤1.5 times the upper limit of reference values (≤3 times for patients with the Gilbert syndrome); and (c) creatinine clearance rate ≥30 mL/minute.

The main exclusion criteria were as follows: (i) patients who received any cell therapy including but not limited to natural killer cell, cytokine-induced killer cell, dendritic cell, and cytotoxic T lymphocyte cell therapy within 28 days prior to the first administration in this trial; (ii) patients positive for human immunodeficiency virus or chronic liver disease other than chronic HBV, such as autoimmune hepatitis, alcoholic liver disease, nonalcoholic fatty liver disease, and drug-induced liver disease; (iii) pregnant or lactating women; and (iv) patients with a history of allergic reactions to blood products or other investigational products. Patient baseline characteristics are summarized in Supplementary Table S1.

**Figure 1.**
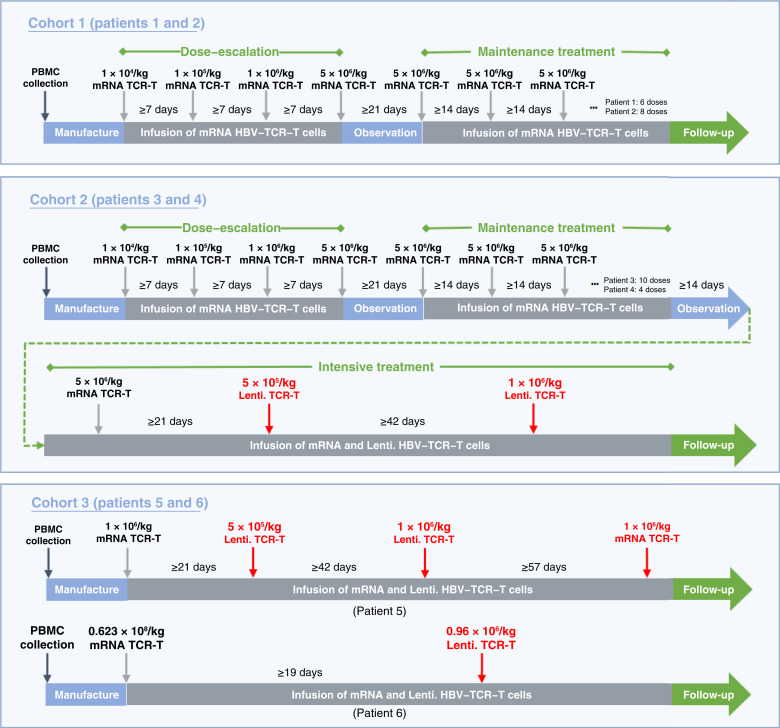
Schematic diagram of the infusion dosage and schedule for HBV–TCR–T cell treatment. Lenti., lentiviral.

The detailed research plan is shown in Fig. 1. To maximize patient safety, we conducted the study in three phases, sequentially enrolling patients into cohort 1, cohort 2, and cohort 3 with adequate safety evaluation of each individual patient prior to the enrollment of the next cohort. The treatment regimens received by each patient are shown in Supplementary Table S2.

#### Cohort 1 (patients 1 and 2)

Patients 1 and 2 were treated with four escalating doses of 1 × 10^4^/kg to 5 × 10^6^/kg mRNA–HBV–TCR–T cells via intravenous infusion at 7-day intervals. Safety observation (≥21-day intervals, only after confirming acceptable safety and tolerability were patients advanced to the next treatment cycle) was followed by multiple 5 × 10^6^/kg mRNA–HBV–TCR–T cell intravenous infusions with at least 14 days between each treatment.

#### Cohort 2 (patients 3 and 4)

The dosing regimen of dose–escalation stage and maintenance treatment stage for patients 3 and 4 was the same as for patients 1 and 2 in cohort 1. On this basis, patients 3 and 4 received additional intravenous infusions—1 dose of 5 × 10^5^/kg and 1 dose of 1 × 10^6^/kg lenti-HBV–TCR–T cells.

#### Cohort 3 (patients 5 and 6)

Patients 5 and 6 received a single dose of 1 × 10^6^/kg mRNA–HBV–TCR–T cells followed by 1 × 10^5^/kg to 1 × 10^6^/kg lenti-HBV–TCR–T cell treatment.

For patients of cohorts 1 and 2, the dosing and number of treatments in the maintenance phase were determined based on the number of TCR–T cells that could be manufactured from each patient’s peripheral blood collected prior to treatment. For patients of cohorts 2 and 3, patients received an initial intravenous infusion of 5 × 10^5^/kg (±25%) lentiviral HBV–TCR–T cells. During subsequent 28-day observation intervals, peripheral blood was monitored for HBV–TCR–T cell concentration. When circulating HBV–TCR–T cell levels fell below 1%, patients received a second intravenous infusion of 1 × 10^6^/kg (±25%) lentiviral HBV–TCR–T cells.

The progressive three-cohort structure was implemented with patient safety as the paramount consideration based on several critical factors. In cohort 1, we first assessed mRNA–HBV–TCR–T cell therapy alone specifically because its transient expression profile (≤7 days) offered a built-in safety mechanism—if unexpected toxicities occurred, they would theoretically be time-limited. Having established the safety of this approach, cohort 2 then investigated lentiviral TCR-T therapy, which provided more durable receptor expression but carries greater potential risks. Finally, cohort 3 combined both modalities at reduced doses based on safety data from the previous cohorts, aiming to further improve safety and optimize the therapeutic window. This stepwise approach allowed us to make real-time protocol adjustments and implement additional safety monitoring as needed while still gathering valuable data across multiple therapeutic strategies within a single-trial framework.

### Manufacturing of HBV–TCR–T cells

**Figure 2.**
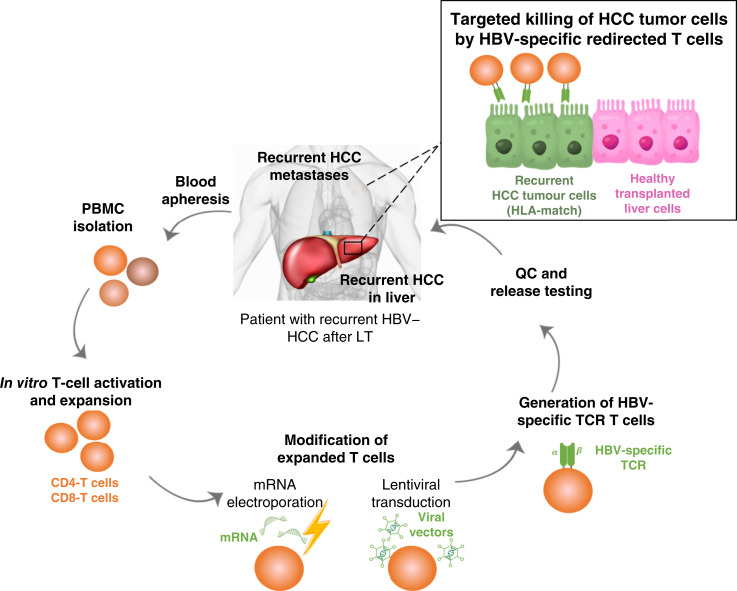
Schematic representation of the production workflow of HBV–TCR–T cells. QC, quality control.

Whole blood from patients was harvested and sent to a GMP-like cell therapy laboratory, in which peripheral blood mononuclear cells (PBMC) were isolated, T cells were activated and expanded, and either mRNA electroporation or lentiviral transduction was performed before harvesting the final cell product (Fig. 2). Briefly, PMBCs were resuspended at 2 × 10^6^ cells/mL in AIM-V medium supplemented with 5% CTS Immune Cell Replacement (Thermo Fisher Scientific), followed by activation and expansion with 600 IU/mL IL-2 (Shandong Quangang) and 50 ng/mL anti-CD3 (OKT-3; Miltenyi Biotec; RRID: AB_2904535).The mRNA or DNA encoding the specific TCR was introduced into the T cells by mRNA electroporation or lentiviral transduction to generate mRNA–HBV–TCR–T cells or lenti-HBV–TCR–T cells, respectively. For the manufacturing of mRNA–HBV–TCR–T cells, T cells were expanded for 8 to 10 days prior to mRNA electroporation for the delivery of mRNA encoding HBV-specific TCR. Electroporated T cells were cultured overnight with 100 IU/mL IL-2 and harvested the following day. For the production of lenti–HBV–TCR–T cells, 50 ng/mL anti-CD28 (BioGems; RRID: AB_2892106) was added to the cell culture. Lentiviral vector supernatant was added after 24 hours of cell culturing at multiplicity of infection of 5. After 48 hours, the cells were resuspended, and cell count was performed. The cell cultures were split and expanded in culture vessels, and AIM-V medium supplemented with 5% CTS Immune Cell Replacement and 600 IU/mL IL-2 was added on alternate days for 7 days. mRNA or lenti–HBV–TCR–T cells were harvested by centrifugation for 5 minutes at 427 *g*, washed twice in saline (containing 5 g/L human albumin), and resuspended in 60 mL of the same solution. A sample of HBV–TCR–T cells was collected for quality control and release testing. After the release test was passed, HBV–TCR–T cells were transported to the clinical center and administered to patients via intravenous infusion.

### Study endpoints and methods

The primary endpoint was to evaluate the safety of HBV–TCR–T cell therapy in patients with HBV–HCC with tumor recurrence after LT by assessing the incidence rate of adverse events (AE) related to HBV–TCR–T cell treatment. AEs were assessed according to the Common Terminology Criteria for Adverse Events. Moreover, peripheral blood samples were collected after each HBV–TCR–T cell infusion to monitor the changes of ALT, AST, TBIL, and albumin (ALB). Its aim was to assess for potential liver damage after HBV–TCR–T cell treatment. To evaluate the systemic inflammatory response, Luminex liquid chip technology was used to measure the serologic level changes of chemokines and cytokines, including CXCL-9, CXCL-10, IL-6, IL-18, and TNF-α.

The secondary endpoint was to assess the antitumor efficacy of HBV–TCR–T cell therapy by assessing the objective response rate by CT scan according to RECIST v1.1 criteria. CT scan restaging was conducted at 1, 3, 6, 9, and 12 months after initiation of TCR-T treatment and continued at similar intervals until radiographic evidence of tumor progression. Progression-free survival (PFS) was defined as the time from the start of study treatment until objective tumor progression or death depending on study protocol. Moreover, the peripheral blood from patients was collected at indicated time points, and PBMCs were isolated to evaluate the function of HBV–TCR–T cells *in vivo*. PBMCs were stimulated with HBV peptides and cultured for 10 days *in vitro* to stimulate antigen-specific HBV–TCR–T cell expansion. ELISpot assays were performed to detect the number of active HBV–TCR–T cells secreting IFN-γ. All patients were followed up monthly during the first 6 months after the end of treatment and thereafter every 3 months for 2 years.

Demographic characteristics, including age, gender, race, height, weight, disease information, and physical condition, were recorded. All patients who received HBV–TCR–T cells were evaluated for safety, which included the incidence and severity of HBV–TCR–T cell infusion-related AEs. AEs were evaluated using the NCI Common Terminology Criteria for Adverse Events. Routine analyses of blood, urine, biochemical parameters (liver and kidney function and electrolytes), coagulation function, myocardial enzymes, and C-reactive protein were performed as safety assessments. Other laboratory examinations included serum α-fetoprotein, HBsAg, and HBV DNA quantification. Vital signs such as heart rate, respiration, body temperature, and blood pressure after HBV–TCR–T cell treatment were monitored.

### Statistical analysis

Descriptive statistics were used to analyze changes in vital signs and related laboratory test results from the baseline values. All clinically significant safety-related AEs that deviated from the baseline data are shown in tabular form. Adverse effects were coded and listed by Medical Dictionary for Regulatory Activities coding system. Continuous variables were analyzed using descriptive statistics, including mean, SD, median, minimum, and maximum values. A *P* value < 0.05 was considered statistically significant.

### Data availability

The data generated in this study are not publicly available due to the risk of compromising patient privacy and consent but are available upon reasonable request from the corresponding author.

## Results

### Patient characteristics

From May 8, 2018, to July 5, 2019, a total of seven patients were enrolled. One withdrew before treatment initiation because of HCC deterioration. A total of six patients (four males and two females) received treatment. The median age of the six patients was 45 years (ranging from 26 to 57 years; Supplementary Table S1). All six patients underwent LT because of HBV-related HCC and were on immunosuppressant medication. The median time of tumor recurrence following LT was approximately 7.85 months. Patients 2, 5 and 6 received local regional and systemic treatment including transcatheter arterial chemoembolization, microwave ablation, and lenvatinib, respectively. All patients were on HBV antiviral treatment throughout the study. One patient was at Barcelona Clinic Liver Cancer (BCLC) stage B, whereas the remaining five patients were at stage C. These patients did not receive any other antineoplastic therapies during the study. Patient 2 died from respiratory/circulatory failure caused by metastatic tumors and cancer cachexia on day 210 after the last infusion. Patients 3, 4, 5, and 6 exited the trial on days 118, 54, 83, and 160 because of the initiation of new antineoplastic treatments, respectively.

### Safety assessment

All AEs observed in this trial, along with their severity grading, are listed in Table 1. Two patients experienced grade 1 to 2 leukopenia, whereas three had grade 1 to 2 elevations in creatine kinase. Other reported AEs during the study treatment included thrombocytopenia, upper respiratory tract infection, insomnia, and needle-like tingling in the chest—commonly observed in patients receiving immunosuppressants and anti-HBV nucleotide analogs following LT for HBV–HCC. Therefore, attributing these AEs specifically to the study treatment remains challenging. Additionally, the grade 2 upper respiratory tract infection in patient 1 may be linked to immune suppression, as suggested by the presence of leukopenia. Furthermore, considering that TCR expression lasts up to 3 days, as previously reported because of the mRNA electroporation delivery method (23), it is also difficult to attribute AEs occurring more than 2 to 3 days after mRNA–HBV–TCR–T cell infusion solely to the T-cell therapy. These events may have been influenced by concomitant medications or the patient’s underlying HCC condition.

**Table 1.** Possible adverse effects associated with HBV–TCR–T cell treatment.

Patient	Adverse effect	CTCAE grade
1	Leukopenia; Thrombocytopenia; Upper respiratory tract infection	1 1 2
2	\	\
3	Leukopenia; Increase in creatine kinase	2 2
4	Increase in creatine kinase	1
5	Insomnia; Increase in creatine kinase; Needle-like chest pain	1 2 2
6	\	\

Abbreviation: CTCAE: Common Terminology Criteria for Adverse Events. “\” indicates “patients without complications.”

**Figure 3.**
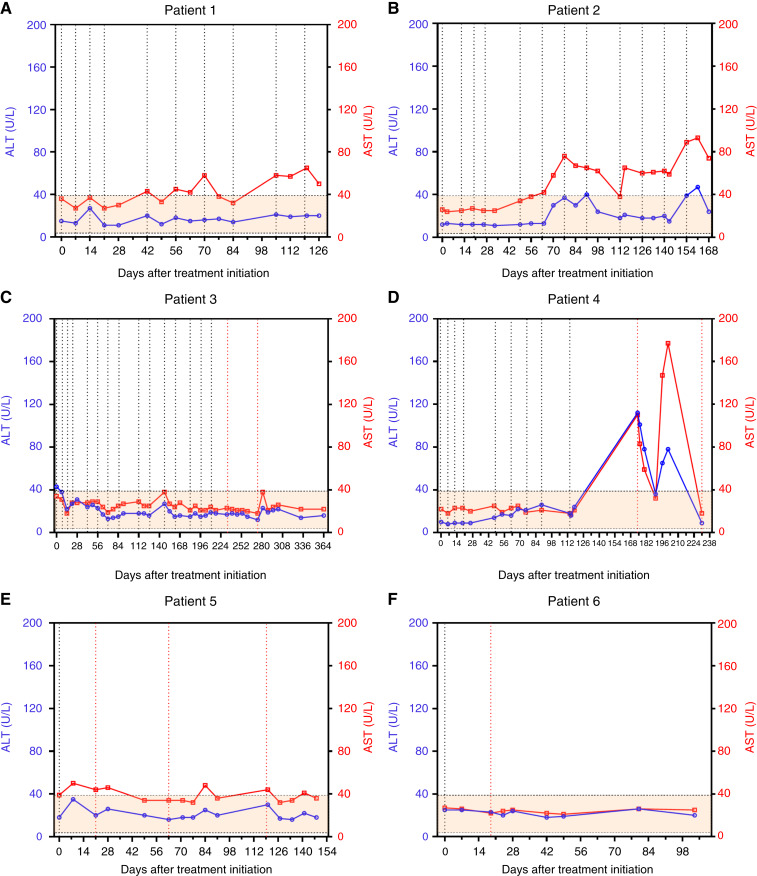
**A–F,** Changes in liver injury markers (ALT and AST) at different time points during HBV–TCR–T cell treatment. The shaded square area in the graph represents the reference range for each marker. The black dashed line indicates the time points of mRNA–HBV–TCR–T cell infusion, and the red dashed line indicates the time points of lenti-HBV–TCR–T cell infusion.

Notably, all AEs were transient and resolved without intervention. No AEs occurred in patients 2 and 6. Furthermore, serum levels of ALT, AST, TBIL, and ALB did not deviate significantly from the reference range (Fig. 3; Supplementary Fig. S2), indicating that none of patients developed immune rejection after HBV–TCR–T cell therapy. Patient 4 specifically experienced a mild elevation in liver enzymes (ALT 112 U/L and AST 110 U/L) after the first infusion of lentiviral HBV–TCR–T cells, and the liver enzymes returned to reference levels completely within 15 days without the use of hepatoprotective drugs. However, after 10 days, patient 4 experienced a mild elevation in liver enzymes again (ALT 78 U/L and AST 177 U/L). Within this timeframe, the patient developed an infection, which was considered related to infection-induced organ dysfunction. During the treatment period, patients 4 and 5 exhibited transient decreases in ALB levels. However, as shown in Supplementary Fig. S2J and S2K, which depict the time points of HBV–TCR–T cell infusion and ALB level trends, this decline could not be definitively attributed to TCR–T cell treatment.

**Figure 4.**
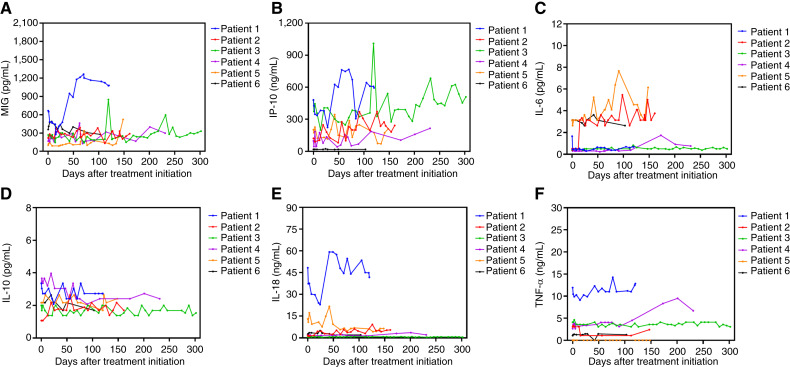
**A–F, **Changes in inflammatory cytokine levels at different time points during the HBV–TCR–T cell treatment. MIG, monokine induced by gamma interferon.

None of the patients experienced serious AEs such as cytokine release storm or immune effector cell–associated neurotoxicity syndrome during the study treatment and follow-up period. This is supported by the stable levels of inflammatory chemokines (CXCL-9 and CXCL-10) and proinflammatory cytokines (IL-6, IL-18, and TNF-α) observed throughout the treatment phase (Fig. 4). These findings provide strong evidence of the safety and tolerability of the treatment protocol. Taken together, these data indicated that either mRNA–HBV–TCR–T cells alone or in combination with lenti-HBV–TCR–T cell therapy were safe and well-tolerated in patients with recurrent HBV–HCC after LT.

### HBV–TCR–T cell function *in vivo*

**Figure 5.**
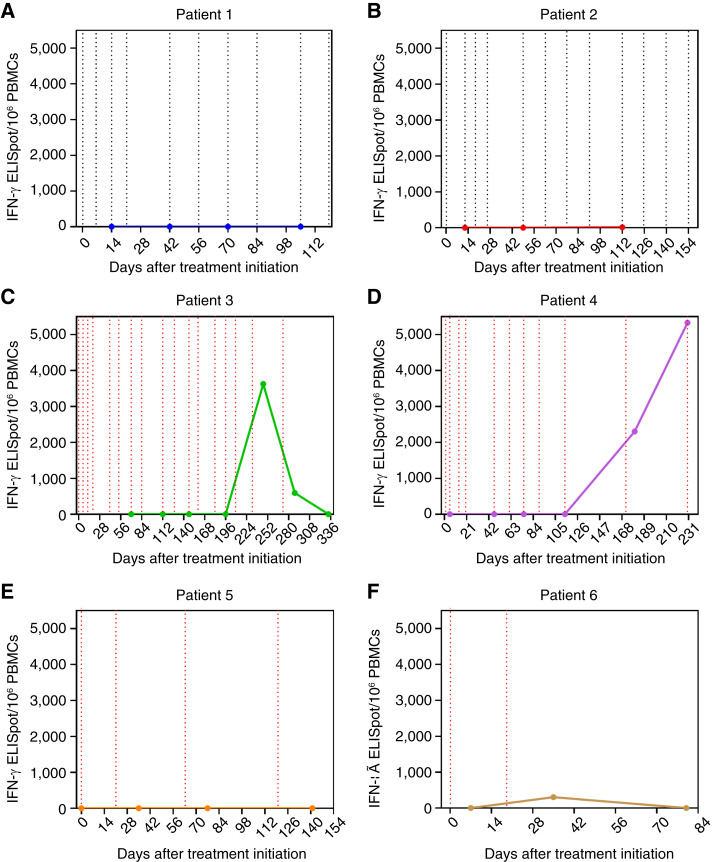
**A–F, **Activity of HBV–TCR–T cells in peripheral blood at various time points. The black vertical dashed lines indicate the infusion time points for mRNA–HBV–TCR–T cells, and the red vertical dashed lines indicate the infusion time points of lenti-HBV–TCR–T cells.

As shown in Fig. 5A and B, no IFN-γ–positive T cells were detected in patients 1 and 2, who received only mRNA–HBV–TCR–T cell infusion. This is likely due to the transient expression nature of mRNA–HBV–TCR, losing their HBV-specific function 72 hours after infusion (17). Similarly, in the combination therapy cohort (patients 3 and 4 and patients 5 and 6), no IFN-γ–secreting T cells were detected during the period when only mRNA–HBV–TCR–T cells were administered (Fig. 5C–F). However, except for patient 5, IFN-γ–positive HBV–TCR–T cells could be detected and maintained up to 61 days after infusion of lenti-HBV–TCR–T cells, as lentiviral transduction resulted in long-term stable expression of HBV antigen–specific TCR in T cells (Fig. 5C–F).

### Efficacy evaluation

The median PFS time and the size of target tumor lesions of each patient are shown in Table 2 and Supplementary Fig. S1, respectively. Patients 1 and 2, who received mRNA–HBV–TCR–T cell treatment alone, showed tumor progression, whereas all patients in the combination therapy group (patients 3 and 4 and patients 5 and 6) showed tumor stabilization.

**Table 2.** Summary of patient response to HBV–TCR–T cell treatment.

Patient	Therapeutic effect	PFS (months)	Status
1	PD	1.87	Alive
2	PD	2.77	Death
3	SD	7.20	Alive (off study)
4	SD	4.47	Alive (off study)
5	SD	7.47	Alive (off study)
6	SD	7.60	Alive (off study)

Data cutoff: April 30, 2020.

Abbreviations: PD, progressive disease; SD, stable disease.

Patients treated with mRNA-transfected HBV–TCR–T cells had a median PFS time of 2.32 months (range, 1.87–2.77 months), whereas the median PFS was 7.34 months (range, 4.47–7.60 months) for the combination therapy group. These data suggested that the combination therapy could be a promising therapeutic option for recurrent HCC following LT. Additionally, our data indicate that lenti-HBV–TCR–T cell therapy may be more tolerant to the long-term concomitant use of immunosuppressive drugs, which are clinically administered to prevent graft rejection after organ transplantation. In contrast, mRNA-transfected HBV–TCR–T cells seem to be more sensitive to immunosuppressive drugs, which could impair the function of infused T cells and limit their clinical efficacy.

Among the two combination groups (cohorts 2 and 3) with different dosing regimens, we observed a reduction in target tumor diameter in patients 5 and 6 (Supplementary Fig. S1). Notably, patient 6 had the longest PFS time, and the CT images showed that the target tumor size remained stable on day 179 after the first dose of HBV–TCR–T cell infusion (Supplementary Fig. S1F).

## Discussion

Immunotherapy strategies, including immune checkpoint inhibitors and autologous immune cell therapy, have been successfully approved to treat cancer, first approved by the US FDA. Particularly, clinical trials involving immune checkpoint inhibitors such as PD-1/PD-L1 inhibitors have demonstrated promising results in the treatment of primary HCC. However, this approach may pose risks for organ transplant recipients, as it activates the immune response throughout the body, potentially leading to various immune-mediated AEs and unexpected inflammation or even graft rejection (24, 25). Rejection following PD-1/PD-L1 blockade was associated with activation of cellular immunity through CD8^+^ effector cells and downregulation of regulatory T cells. Consequently, LT patients with HBV–HCC relapses are currently not considered for therapeutic approaches involving T cell–mediated immune activation. Furthermore, CAR-T therapy is less effective in solid tumor clinical trials. Therefore, addressing postoperative recurrence in this patient population remains an urgent challenge.

In this study, two types of HBV–TCR–T cells—mRNA–HBV–TCR–T cells and lenti-HBV–TCR–T cells—were studied. The six patients enrolled received 2 to 17 infusions, with a total of 9 × 10^7^ to 3.6 × 10^9^ cells of HBV–TCR–T cell therapies. None of them experienced any serious treatment-related AEs during the trial. No cytokine release syndrome and neurotoxicity were observed in this study, demonstrating a favorable safety profile of HBV–TCR–T cell therapy. Importantly, these autologous modified T cells can specifically target HBV–HCC tumor cells without damaging the hepatocytes from the transplanted liver because of HLA mismatch in patients who underwent LT. This suggests that autologous HBV–TCR–T cell therapy is a safe treatment approach for this patient group, which lacks effective standard therapies. Theoretically, the majority of HBV-associated tumor cells express HBV antigen peptides, whereas donor-derived transplanted liver cells are HBV-negative. As part of standard care for after LT, patients with HBV–HCC typically receive anti-HBV drugs and HBV immunoglobulin to reduce the likelihood of HBV infection in the transplanted livers (15, 26). Moreover, even if HBV infection occurs, the liver is an immunotolerant organ and does not require strict HLA matching between the donor and recipient (24). As a result, autologous HBV–TCR–T cells are unlikely to recognize HBV antigen–HLA complexes in the transplanted liver due to HLA mismatch, thereby minimizing the risk of graft-related liver toxicity (15).

The initial use of mRNA electroporation for TCR delivery offers durable therapeutic effects while minimizing safety risks. Administering mRNA–HBV–TCR–T cell therapy allows for close monitoring of potential AEs associated with TCR expression, which can help guide subsequent treatment decisions with lenti-HBV–TCR–T cell therapy. From scalability and manufacturing perspectives, the use of mRNA technology also allows for the ease of indication expansion. By cloning different mRNA sequences encoding distinct TCRs into a plasmid vector, we can customize the TCR repertoire for various therapeutic applications or disease targets. Simply replacing the cloned TCR mRNA sequence enables the expressed TCR to recognize and bind to a different antigen, allowing for rapid retargeting of the therapy to address diverse diseases or specific immune responses. This flexibility streamlines the development and optimization of TCR-based therapies.

As for primary HCC, the target lesion is typically located in the liver, which is a critical organ. Due to its transient expression nature of mRNA, mRNA–HBV–TCR–T cell therapy perfectly fits this scenario. The safety and efficacy of mRNA–HBV–TCR–T cell therapy in patients with primary HBV–HCC have been demonstrated in studies. However, in the posttransplantation setting, the routine concomitant administration of immunosuppression regimens can compromise the efficacy of T-cell therapy. After transplantation, patients receive tacrolimus alone or in combination with mycophenolate mofetil throughout their life. These drugs were designed to broadly suppress the T cell–mediated immune responses and prevent graft rejection. It has been demonstrated that even short-term exposure to these drugs can significantly affect the function and migration of T cells, negatively affecting the clinical efficacy of T-cell therapy (16).

In this study, we demonstrated that the combination of mRNA–HBV–TCR–T cells and lenti-HBV–TCR–T cells yielded promising therapeutic outcomes. Four patients received lentiviral-transduced HBV–TCR–T cells (4–15.5 × 10^7^ cells) following treatment with mRNA–HBV–TCR–T cells. No graft rejection was observed, and all these four patients achieved disease stabilization. However, sorafenib (first-line HCC therapy) showed efficacy in only a minority of LT recipients with HBV-related HCC recurrence according to stable disease status. A 2015 study showed that the disease remains stable in most cases (46.5%), whereas a significant number of patients (37%) experience disease progression (27). The median PFS time of the four patients who received combination therapy (mRNA–HBV–TCR–T cells + lenti-HBV–TCR–T cells) was 7.34 months, surpassing the 6.21-month median PFS reported in a 2015 analysis from Queen Mary Hospital in Hong Kong, which assessed the use of sorafenib in patients with HCC recurrence after LT (28). A 2017 study at the University Health Network reported a median time to progression of 4.3 months among 24 patients with post-LT HCC recurrence treated with sorafenib (29). Additionally, a more recent retrospective study across 11 oncology centers in Turkey evaluated sorafenib and regorafenib in 73 patients with the same indication (30). The median PFS with sorafenib, administered as first-line treatment, was 5.6 months, whereas regorafenib, administered as second-line treatment, achieved a median PFS of 5.9 months. These data underscore the potential of combining mRNA-HBV–TCR–T cells and lenti-HBV–TCR–T cell therapy for this indication, in which effective therapies are currently lacking. Notably, reducing the total HBV–TCR–T cell dose did not compromise therapeutic efficacy, underscoring the feasibility and efficacy of this combination approach.

In patients receiving only mRNA–HBV–TCR–T cell therapy, immunosuppressive drugs may impair the function of the mRNA-TCR-T cells, thereby limiting their therapeutic effectiveness. This observation is consistent with a previously reported study in the same indication (20). To overcome the immunosuppressive effects of immunosuppression regimens, immunosuppressive drug–resistant armored HBV-redirected TCR T cells have been developed by incorporating mRNA encoding HBV-specific TCR along with mutated forms of calcineurin subunit B and inosine-5′-monophosphate dehydrogenase, both of which are targets of commonly used immunosuppressive drugs (16). The transient nature of mRNA expression limits the modified T cells’ functional lifespan to approximately 3 to 5 days *in vivo*, after which they revert to their original specificity and regain sensitivity to immunosuppressive drugs. Exploration using immunosuppressive drug–resistant armored HBV-redirected TCR T cells is warranted in future studies.

Whereas lentiviral transduction enables stable TCR expression, enhancing long-term efficacy, its use raises safety considerations, particularly regarding secondary oncogenesis and prolonged TCR expression, which could lead to uncontrolled immune-related toxicities. Additionally, the need for lymphodepleting chemotherapy prior to TCR–T cell infusion presents further safety concerns, including cytopenia, increased infection risk, and delayed immune reconstitution (21), which are particularly relevant in posttransplant patients already receiving immunosuppressive therapy. However, mRNA–HBV–TCR–T cells do not require prior lymphodepletion, avoiding these side effects and making them a safer option in this context. In the setting of recurrent HBV–HCC after LT, the presence of an HLA-mismatched liver graft provides a degree of specificity, as HBV–TCR–T cells selectively target HBV-infected tumor cells rather than normal liver tissue, thereby mitigating the risk of graft-related toxicity. This targeted approach suggests that lentiviral-transduced HBV–TCR–T cells remain a viable option for tumor PTR despite these challenges.

Overall, our findings suggest that combining mRNA-transfected and lentiviral-transduced HBV–TCR–T cells not only maintains a favorable safety profile but also preliminarily shows promising antitumor efficacy for treating recurrent HBV–HCC after LT, in which lifelong administration of immunosuppression drugs is required. Importantly, the HLA mismatch between the transplanted liver and the recipient’s immune system reduces the likelihood of on-target, off-tumor toxicity, reinforcing the safety of this approach compared with its use in primary HCC.

## Supplementary Material

Figure S1 Figure S1

Figure S2 Figure S2

Supplementary Data1 Supplementary Data
