# Supplementary material for: HBV-Specific TCR–T Cell Therapy Combining mRNA Electroporation and Lentiviral Transduction: Treatment Regimen for Recurrent HBV-Related HCC after Liver Transplantation
Source: Clin Cancer Res. 2025 Jul 24;31(18):3886–96. doi: 10.1158/1078-0432.CCR-25-1245 (PMC12434392; doi:10.1158/1078-0432.CCR-25-1245)
Supplement: Figure S1 [file ccr-25-1245_figure_s1_suppfs1.pptx]

## Slide 1
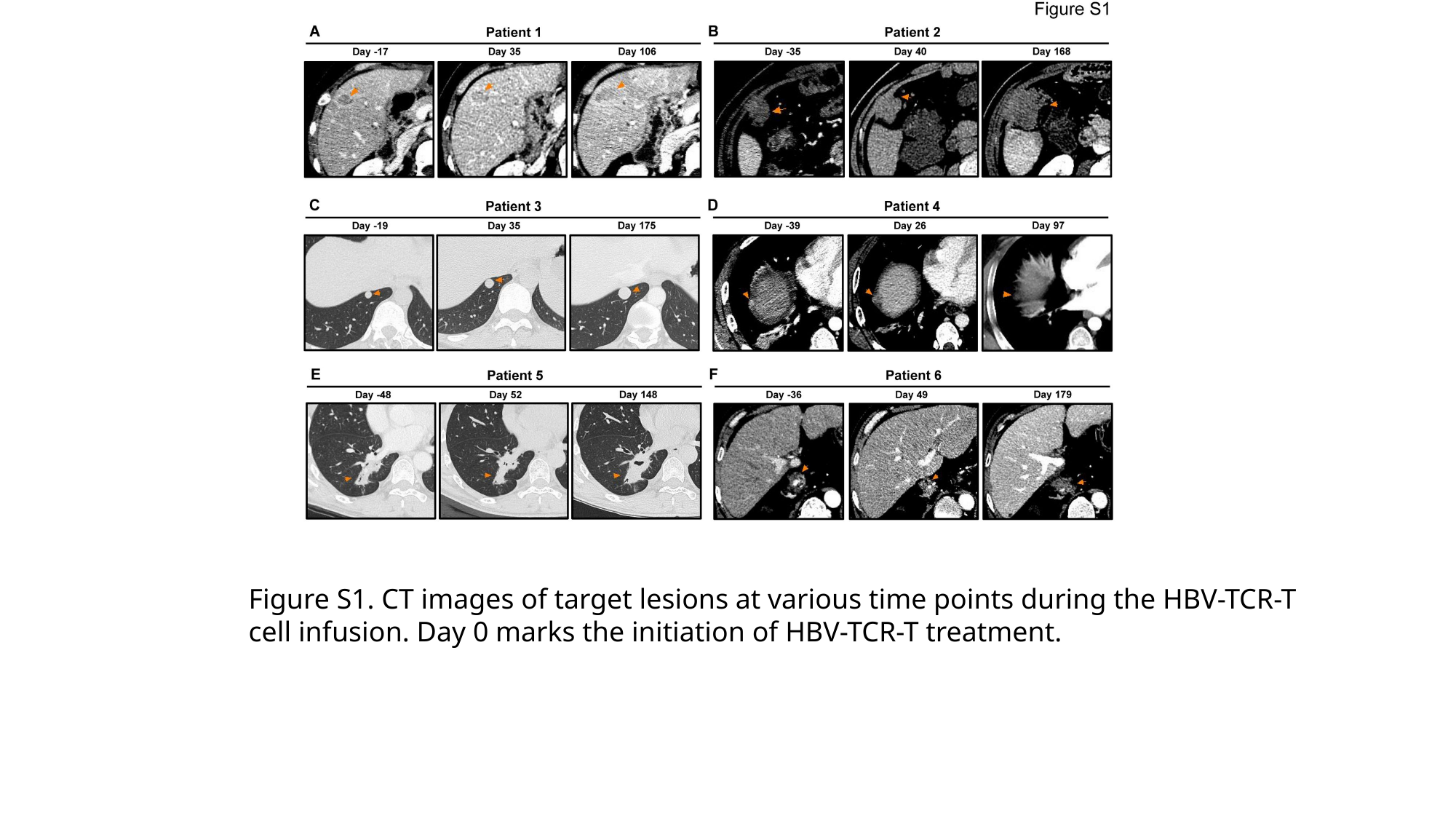

Figure S1. CT images of target lesions at various time points during the HBV-TCR-T cell infusion. Day 0 marks the initiation of HBV-TCR-T treatment.
