# Supplementary material for: HBV-Specific TCR–T Cell Therapy Combining mRNA Electroporation and Lentiviral Transduction: Treatment Regimen for Recurrent HBV-Related HCC after Liver Transplantation
Source: Clin Cancer Res. 2025 Jul 24;31(18):3886–96. doi: 10.1158/1078-0432.CCR-25-1245 (PMC12434392; doi:10.1158/1078-0432.CCR-25-1245)
Supplement: Figure S2 [file ccr-25-1245_figure_s2_suppfs2.pptx]

## Slide 1
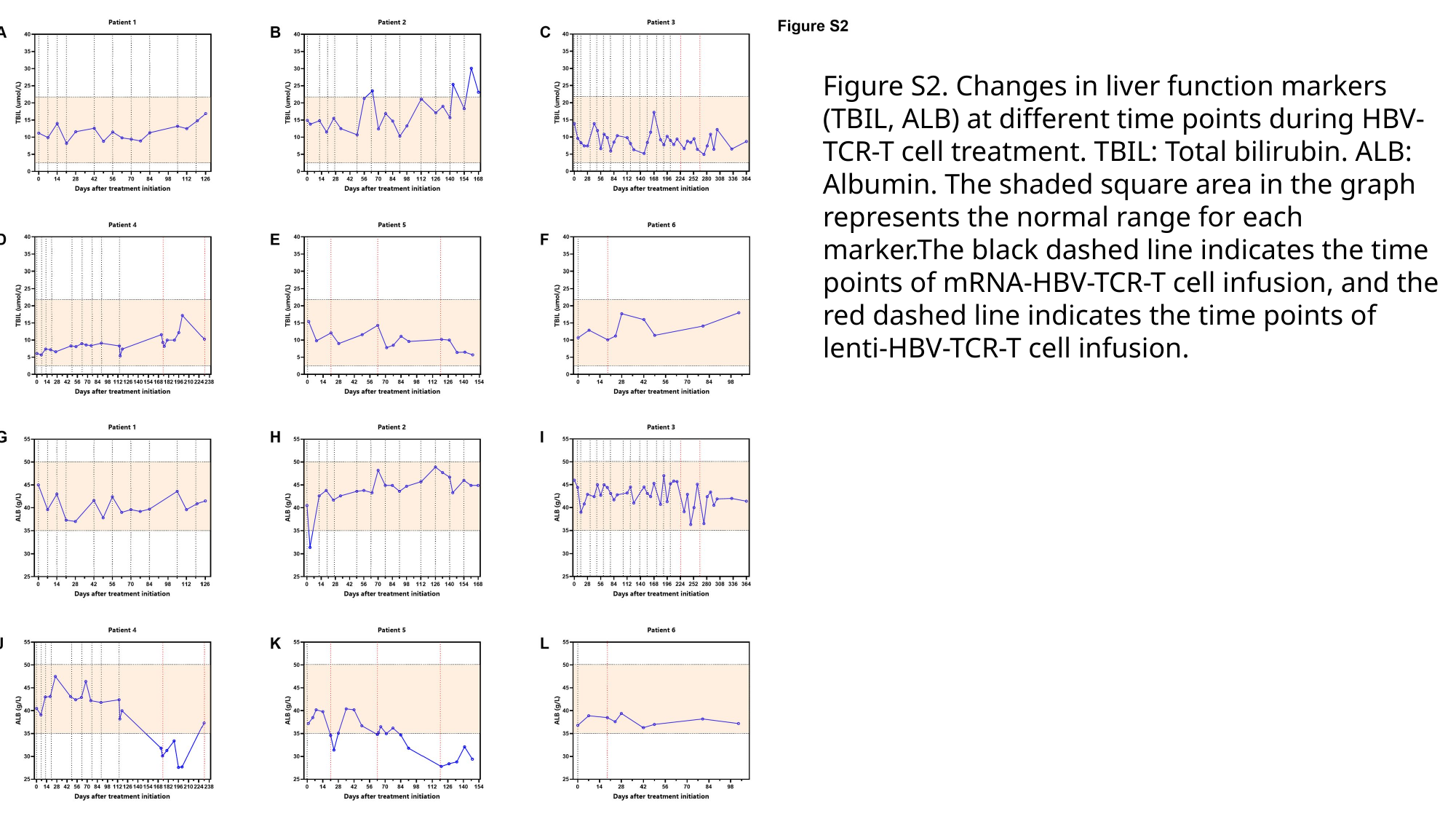

Figure S2. Changes in liver function markers (TBIL, ALB) at different time points during HBV-TCR-T cell treatment. TBIL: Total bilirubin. ALB: Albumin. The shaded square area in the graph represents the normal range for each marker.The black dashed line indicates the time points of mRNA-HBV-TCR-T cell infusion, and the red dashed line indicates the time points of lenti-HBV-TCR-T cell infusion.
