# Supplementary material for: HBV-Specific TCR–T Cell Therapy Combining mRNA Electroporation and Lentiviral Transduction: Treatment Regimen for Recurrent HBV-Related HCC after Liver Transplantation
Source: Clin Cancer Res. 2025 Jul 24;31(18):3886–96. doi: 10.1158/1078-0432.CCR-25-1245 (PMC12434392; doi:10.1158/1078-0432.CCR-25-1245)
Supplement: Supplementary Data1 — Supplementary Data [file ccr-25-1245_supplementary_data1_suppds1.docx]

**Supplementary Data for review**

Table Legends

Table S1. Baseline characteristics of all treated patients.

| **Cohort** | **Patient** | **Age** | **HBsAg serum (IU/ml)** | **Gender** | **Tumor burden** | | | | | **Time to tumor recurrence after LT（month）** | **Treatment received following LT** | **Immunosuppressant drug administered** |
| --- | --- | --- | --- | --- | --- | --- | --- | --- | --- | --- | --- | --- |
|  |  |  |  |  | **Anatomical location of relapses** | **Number** | **Maximum diameter（mm）** | **AFP**  **(ug/L)** | **BCLC stage** |  |  |  |
| 1 | 1 | 49 | 19.99 | Female | Liver | >3 | 16.4 | 1202 | B | 8.7 | \ | FK506 + MMF |
|  | 2 | 53 | Undetectable | Male | Omentum; Liver; Lung | >3 | 29.8 | 790 | C | 5.3 | E7080 | FK506 + MMF |
| 2 | 3 | 41 | 1070.33 | Female | Lung | >3 | 9 | 45 | C | 15.6 | \ | FK506 + SRL + MMF |
|  | 4 | 26 | Undetectable | Male | Diaphragm | 1 | 10 | 5502 | C | 1.2 | \ | FK506 + SRL |
| 3 | 5 | 38 | Undetectable | Male | Lung | >3 | 51 | 1360 | C | 7 | E7080 | FK506 + SRL |
|  | 6 | 57 | Undetectable | Male | Liver; Esophageal lymph node; Inferior vena cava lymph node | >3 | 32 | 2.49 | C | 29.1 | E7080; TACE; MWA | SRL |

Note: E7080: Lenvatinib. TACE: Transcatheter arterial chemoembolization. MWA: microwave ablationFK506: Tacrolimus. SRL: Sirolimus. MMF: Mycophenolate mofetil.

Table S2. Summary of patients receiving HBV-TCR-T cell treatment

| **Cohort** | **Patient** | **Weight (kg)** | **HBV-DNA integration** | **HLA restriction / antigen specificity of HBV-TCR** | **TCR-T**  **cell type** | **Total number**  **(×10^7^)** | **Doses** | **Treatment duration**  **(Days)** |
| --- | --- | --- | --- | --- | --- | --- | --- | --- |
| 1 | 1 | 53.4 | + | B*58:01 / HBsAg | mRNA-TCR-T | 189.7 | 10 | 120 |
|  | 2 | 63.6 | + | A*02:01 / HBsAg | mRNA-TCR-T | 294.8 | 12 | 155 |
| 2 | 3 | 58.0 | + | A*11:01 / HBcAg | mRNA-TCR-T | 350.7 | 15 | 275 |
|  |  |  |  |  | Lenti-TCR-T | 8.9 | 2 |  |
|  | 4 | 84.3 | + | C*08:01 / HBsAg | mRNA-TCR-T | 253.9 | 9 | 231 |
|  |  |  |  |  | Lenti-TCR-T | 13.0 | 2 |  |
| 3 | 5 | 60.8 | + | A*02:01 / HBsAg | mRNA-TCR-T | 6.2 | 1 | 121 |
|  |  |  |  |  | Lenti-TCR-T | 15.5 | 3 |  |
|  | 6 | 81 | + | A*02:01 / HBsAg | mRNA-TCR-T | 5.0 | 1 | 20 |
|  |  |  |  |  | Lenti-TCR-T | 4.0 | 1 |  |

Table S3. Representativeness of Study Participants

| **Cancer condition** | Recurrent HBV-related hepatocellular carcinoma (HBV-related HCC) post-liver transplantation |
| --- | --- |
| **Sex** | The male-to-female ratio ranges from 5:1 to 7:1 for HBV-related HCC. |
| **Age** | HCC tends to occur later in life in Japan, North America and European countries, where the median age of onset is above 60 years. However, in Asia and most African countries, HCC is commonly diagnosed in the age range 30–60 year.The HCC BRIDGE study of 18,031 patients with HCC from 42 sites in 14 countries showed that the mean age at HCC diagnosis was 52 in China. |
| **Ethnicity** | HCC accounts for 90% of primary liver cancer cases. However, the incidence of HCC related to viral liver disease is decreasing in developed countries, and it is counterbalanced by an increasing prevalence of nonalcoholic fatty liver disease (NAFLD) and its related HCC. However, HBV is the major risk factor of liver cirrhosis in eastern countries, where it has its higher prevalence and incidence, and so HBV-related HCC has consequently a higher incidence in Asia. Specifically, the prevalence of HBV infection in the general Chinese population was approximately 5–7.99% in 2018, of which more than 90% were adults older than 20 years. |
| **Geography** | HBV is the primary etiologic agent of HCC. In Asian countries with a high prevalence of chronic HBV infection, HBV-related HCC accounts for at least 80% of HCC. |
| **Other considerations** | Post-transplant recurrence is typically characterized by widespread metastases, involving multiple organs such as the lungs, liver, bones, lymph nodes, and adrenal glands in over 50% of cases. |
| **Overall representativeness of this study** | Overall, the enrolled patients in this study were representative of the target population. Among study participants, the male-to-female ratio was 3:1, with a median age of 45 years (range: 26-57 years), consistent with published epidemiological data. All six patients were HBV-infected Asian individuals, five of whom developed multi-organ tumor metastases post-liver transplantation, exhibiting representative tumor characteristics. |
